# Endomicrobial Community Profiles of Two Different Mealybugs: *Paracoccus marginatus* and *Ferrisia virgata*

**DOI:** 10.4014/jmb.2001.01016

**Published:** 2020-04-02

**Authors:** Polpass Arul Jose, Ramasamy Krishnamoorthy, Pandiyan Indira Gandhi, Murugaiyan Senthilkumar, Veeranan Janahiraman, Karunandham Kumutha, Aritra Roy Choudhury, Sandipan Samaddar, Rangasamy Anandham, Tongmin Sa

**Affiliations:** 1 Department of Agricultural Microbiology, Agricultural College and Research Institute, Madurai, Tamil Nadu Agricultural University, Tamil Nadu, India; 2 Regional Research Station, Vridhachalam, Tamil Nadu Agricultural University, Tamil Nadu, India; 3 Department of Agricultural Microbiology, Tamil Nadu Agricultural University, Coimbatore, Tamil Nadu, India; 4 Department of Environmental and Biological Chemistry, Chungbuk National University, Cheongju 28644, Republic of Korea

**Keywords:** Ecology, mealybug, endomicrobiota, phylogeny, *Paracoccus marginatus*, *Ferrisia virgata*

## Abstract

Comparative analysis of microbial communities associated with these mealybugs revealed differences that appear to stem from phylogenetic associations and different nutritional requirements. This first report on both bacterial and fungal communities associated with these mealybugs provides a preliminary insight on factors affecting the endomicrobial communities.

Insects are the most abundant animals in terrestrial ecosystems and are inhabited by symbiotic microbes that provide beneficial services to their hosts [1]. Mealybugs (Homoptera: Coccoidea: Pseudococcidae) are plant- sucking scale insects affecting agricultural ecosystems and causing damage to more than 300 plant species [2-4]. Examining the ecological interactions of symbiotic microbes in agriculturally important insect-hosts may lead to novel methods of pest control and enhancement of agricultural productivity [5, 6]. Diet and the insect lineage have been proposed to exert different influences on symbiotic microbial diversity [7]. Accordingly, in this study we examined the hypothesis that the mealybug microbial ecology is strongly determined by interaction between phylogenetic constraints and nutritional requirements. To this end, we performed endomicrobial community analysis of two phylogenetically distinct mealybug species that feed concurrently on the same plant yet differ in their choice of feeding site and the processing of the plant-derived diet. 1) Papaya mealybug (PM), *Paracoccus marginatus* Williams and Granara de Willink, feeds frequently on phloem sap and produces large amounts of honeydew [8]. 2) Two-tailed mealybug (TM), *Ferrisia virgata* Cockerell, colonizes around the terminal parts of the plant, infrequently accesses the phloem sap and produces small amounts of honeydew [9]. Based on integrated molecular and morphological data, these two mealybugs have been assigned under different sub-families [10].

Pooled PM and TM samples were created from fifty individuals of each mealybug species collected from a papaya (*Carica papaya*) plant cv. CO8 at Agricultural College and Research Institute, Madurai (9°56'0" N and 78°7'0" E), India. The insects were surface sterilized by rinsing in sterile water, soaking in 70% ethanol and 10% bleach, with three rinses in water, for 60 sec at each step. The surface-sterilized insects were homogenized in liquid nitrogen. Whole DNA was extracted from homogenates using an Insect DNA Purification Kit (Hi-Media, India) according to the manufacturer’s protocol. The extracted DNA was amplified using primers targeting the V3-V4 region of the bacterial 16S rRNA gene (primers, 341F and 518R) and fungal internal transcribed spacer (primers, ITS5F and ITS2R) [11, 12]. Sequencing was performed using the Illumina MiSeq platform at Genotypic Technology, India. Resulted sequence data were deposited in SRA archives under accession number PRJNA522349. The raw reads were processed as described elsewhere [13]. The processing of raw reads clustered at 97% identity revealed 5,881 and 2,417 bacterial operational taxonomic units (OTUs), whereas 258 and 172 fungal OTUs were identified for PM and TM, respectively. The rarefaction curve (Fig. S1) indicated the adequate sampling effort for getting the full extent of taxonomic diversity. It explains how the number of species found in a sample at any given phylogenetic level is strongly affected by the number of sequences analyzed [14]. The Shannon and Simpson diversity indices, and richness estimator (Chao) of bacteria and fungi were relatively higher for PM compared to TM (Table 1). However, the diversity and richness indices of fungi were relatively poor for both the samples compared to the bacterial counterpart. The gut bacterial community had proteobacterial abundance for both of the mealybug species, followed by Actinobacteria and Firmicutes in PM and TM, respectively (Fig. 1A). A previous study of 305 individual insects belonging to 21 taxonomic orders showed that the insect microbiota is dominated by the Proteobacteria and Firmicutes [15]. Moreover, the specific abundance of Proteobacteria further correlates well with bacterial communities of different mealybug species [16-18]. Recently, Iasur-Kruh *et al*. [17] reported that Betaproteobacteria (58%) and Gammaproteobacteria (33%) constituted the proteobacterial community in the vine mealybug. However, this pattern deviated in papaya and two-tailed mealybugs. In papaya mealybug, all the three classes of Proteobacteria (Betaproteobacteria (83.41%), Alphaproteobacteria (7.19%) and Gammaproteobacteria (4.63%) were represented, while Betaproteobacteria is most abundant (97.6%) in two-tailed mealybug (Fig. 1B). At the genus level, PM and TM samples were dominated by the obligate nutritional endosymbionts *Tremblaya,* which has been reported to provide aid in nutrition and detoxification of plant substances [19, 20]. The ITS sequence- based fungal community analysis revealed the occurrence of seven fungal phyla (Fig. 2A). The majority of sequences were affiliated with Ascomycota (78.75%), followed by Zygomycota (4.74%) and Basidomycota (5.91%). Individually, Ascomycota dominated with 75.28% and 91.65% in PM and TM respectively. The phylum Zygomycota and two different unclassified populations were moderately abundant in PM, while it was less abundant in TM. However, Glomeromycota was more abundant in TM rather than in PM. However, among the classified genera, *Cladosporium* showed moderate abundance (8.04%), followed by *Aureobasidium* (5.54%), *Mortierella* (4.61%). Notably, the majority of *Aureobasidium* and *Mortierella* counts were from PM (Fig. 2B). The results of earlier studies using culture-dependent and molecular techniques indicated that vine mealybug, *Planococcus ficus,* largely harbors Ascomycota [18]. *Cladosporium* and *Mycosphaerella*, common environmental fungi, were equally found in both TM and PM. Iasur-Kruh *et al*. [17] found that *Cladosporium* is abundant in vine mealybug reared in lab. This suggests that *Cladosporium* and *Mycosphaerella* are more transient fungal associates acquired via feeding. A recent study using confocal microscopy revealed that a similar fungus, *Beauveria bassiana*, penetrates mealybug *Phenacoccus manihoti* through the legs and mouthparts [21]. Interestingly, several fungal species were found in one mealybug species, while not in the other, though they were sampled from the same plant. For instance, Zygomycota was found in PM, but not in TM. Similarly, Glomeromycota was found in TM, yet it is absent in PM. These differences could be due to selective ecological pressure exerted by the host [18, 22].

Among the symbionts associated with PM and TM, *Tremblaya* is a well-known endosymbiont of mealybugs. Bacterial communities other than *Tremblaya* associated with the PM and TM were retrieved from the taxonomy data and mapped for their metabolic activities using different phenotypic categories in METAGENassist [23]. The analysis was done using the standard settings suggested in the server. The results revealed the occurrence of 15 types of metabolic activities (Fig. 3). Interestingly, abundant bacteria capable of ammonia oxidization, atrazine metabolism, dehalogenation, nitrate reduction, sulfur metabolism, and xylan degradation were found in both mealybug species. The automated metabolic functional mapping of the abundant bacterial community revealed diverse activities, and strongly suggested the role of the bacterial communities associated with both mealybugs in providing nutritional as well as detoxification support to the host insects. For instance, those microbes having the ability to fix and mobilize different nutrients (nitrogen and sulfur) could provide dietary support to the insect host, while others are involved in detoxification of toxic compounds [24]. Difference in the relative abundance of different metabolic functions suggests evolutionary trajectories of the microbial communities, tailored to specific needs of the hosts [24, 25].

In summary, this study disclosed the endomicrobial communities (bacteria and fungi) in two mealybug species, *P. marginatus* and *F. Virgata,* differ in phylogenetic and nutritional characteristics. Despite a lack of sequencing replicates, this study provides a preliminary insight into the relationships between different mealybugs and their microbial communities. Differences among the microbial communities appear to be associated largely with their phylogeny and different nutritional characteristics while they fed on the same plant sap. The super dominant bacteria was *Tremblaya* in both cases, but intra generic diversity was found within this genus in PM. Dynamics of *Tremblaya* in PM, as well as the moderately abundant and rare species in both mealybug species have to be studied under controlled conditions to reveal their biological and symbiotic roles.

## Availability of Data and Materials

The data and analyses from the current study are available from the corresponding author upon reasonable request. The raw reads were deposited in SRA archives and can be accessed by accession number PRJNA522349.

## Supplemental Materials



Supplementary data for this paper are available on-line only at http://jmb.or.kr.

## Figures and Tables

**Fig. 1:**
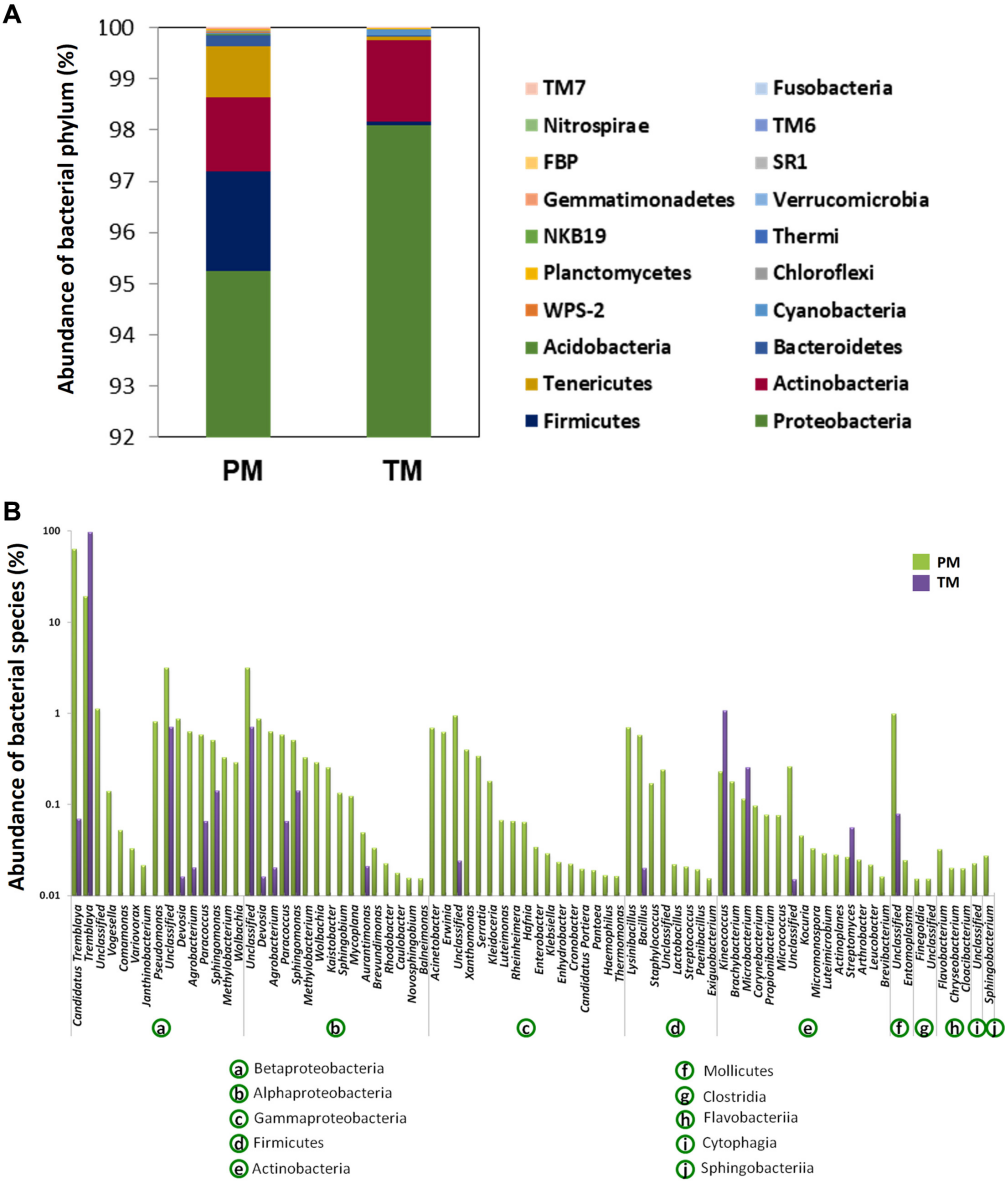
Bacterial community profile decoded by 16S amplicon-metagenomics. Relative abundance of the gut- bacterial lineages (after removing endosymbionts) found in PM and TM at (**A**) phylum level and (**B**) species level.

**Fig. 2:**
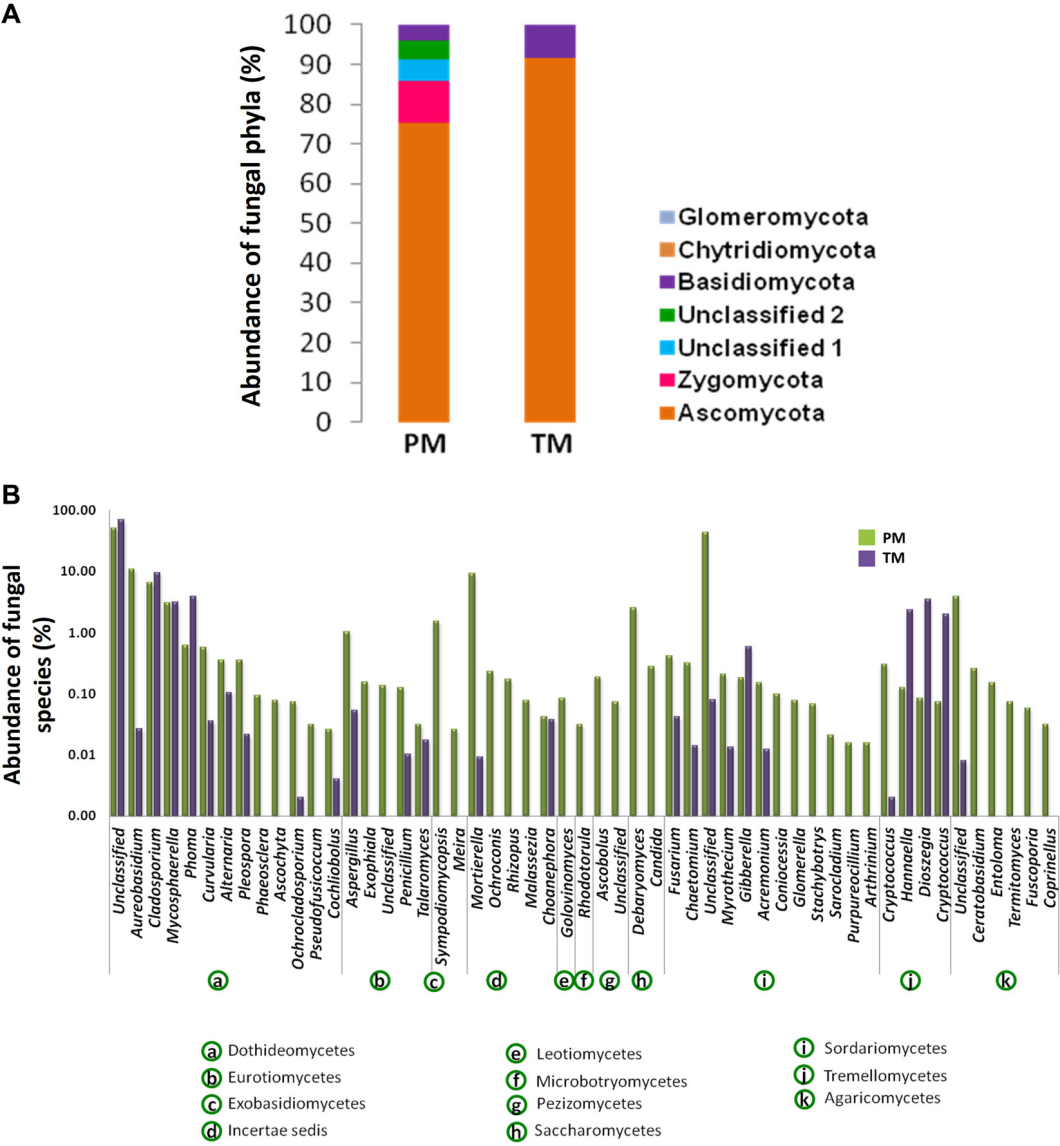
Fungal community profile decoded by 18S (ITS) amplicon-metagenomics. Relative abundance of the gut- fungal lineages (after removing endosymbionts) found in PM and TM at (**A**) phylum level and (**B**) species level.

**Fig. 3:**
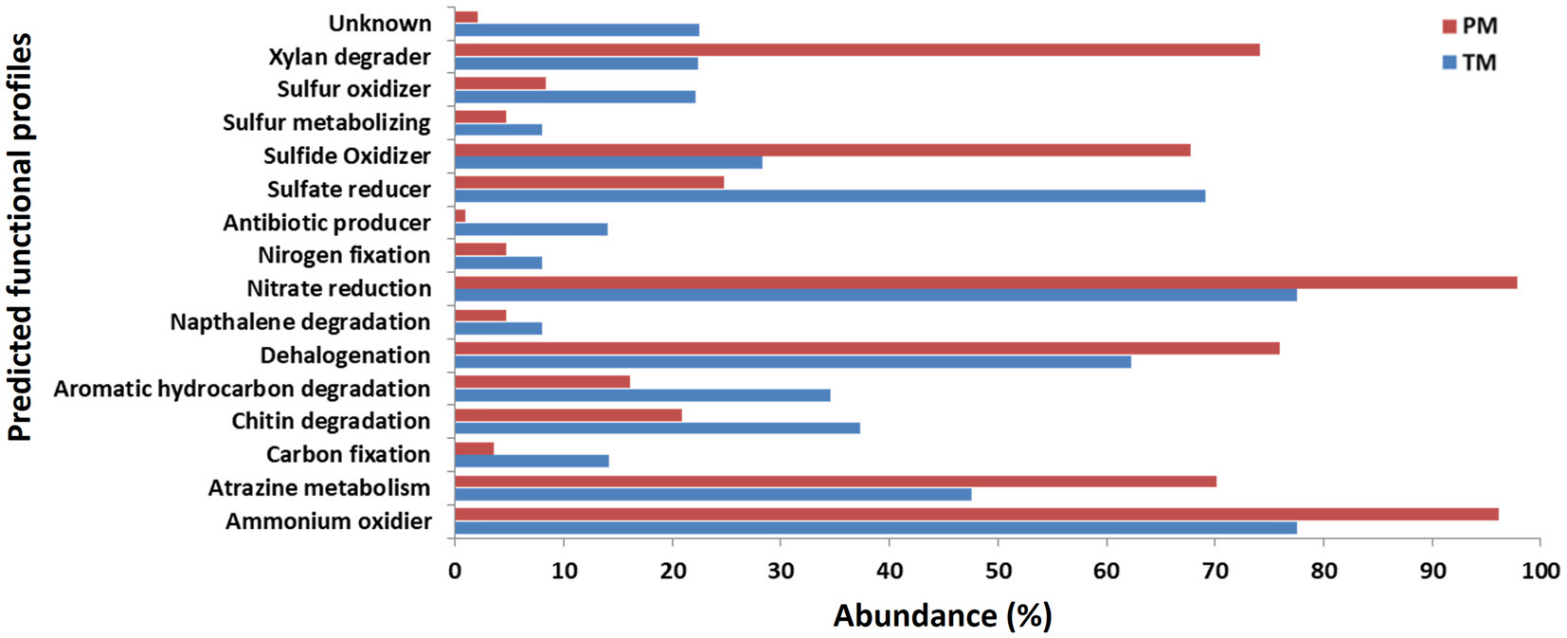
Functional mapping of the bacterial community associated with PM and TM.

**Table 1:** Microbial diversity estimates in the gut of papaya mealybug and two-tailed mealybug.

Domain	Type of mealybug	Number of OTUs	Chao1 (Richness)	Shannon (Diversity)	Simpson (Diversity)
Bacteria	PM	5881	10061.46	3.42	0.64
	TM	2417	4833.76	1.87	0.48
Fungi	PM	258	276	1.30	0.91
	TM	170	172	0.54	0.48
