# Supplementary figures and images for: Endomicrobial Community Profiles of Two Different Mealybugs: Paracoccus marginatus and Ferrisia virgata
Source: J Microbiol Biotechnol. 2020 Apr 2;30(7):1013–7. doi: 10.4014/jmb.2001.01016 (PMC9728186; doi:10.4014/jmb.2001.01016)

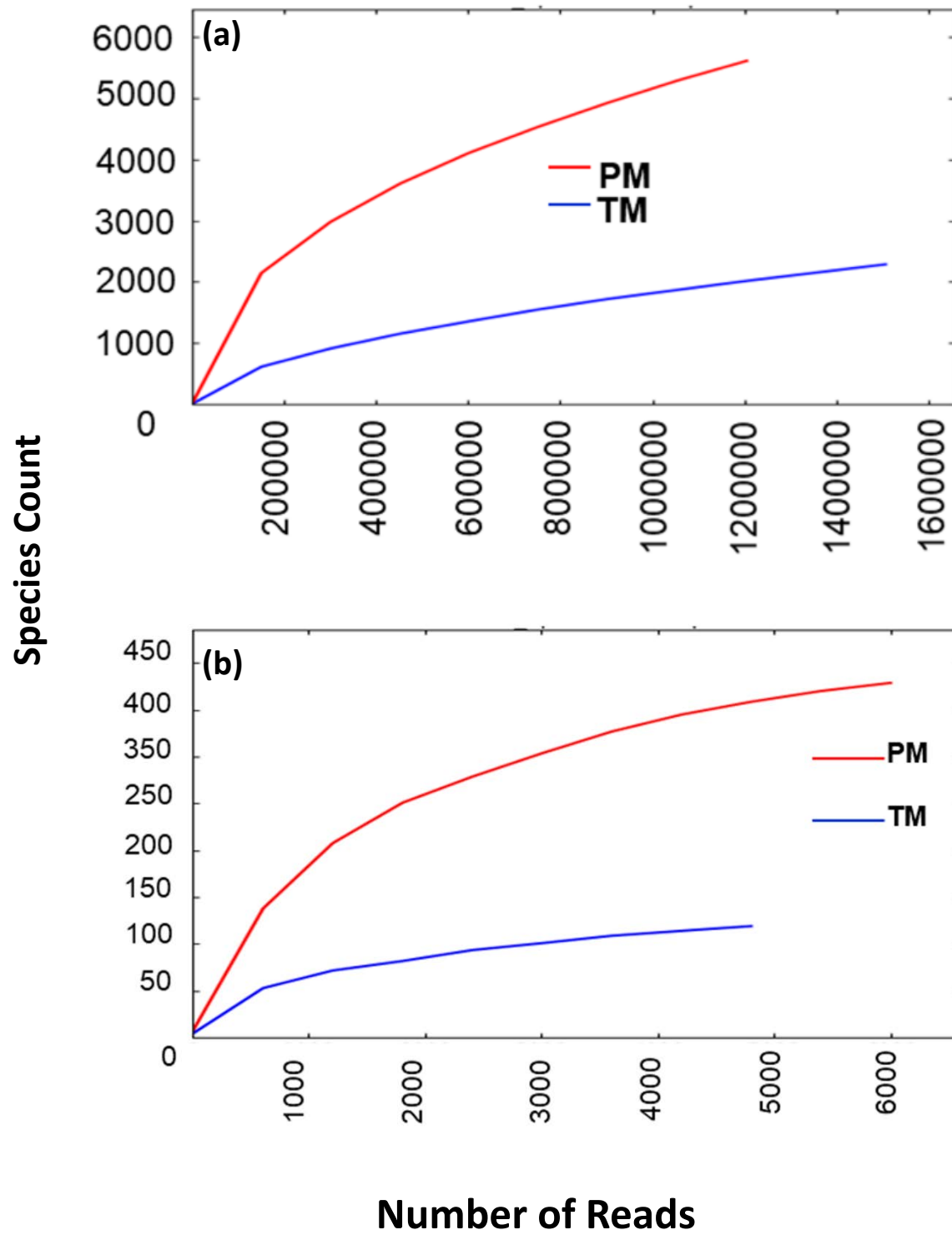

Fig S1: Rarefaction curve of (a) bacterial and (b) fungal communities

Supplement: Supplementary file 1 [file JMB-30-7-1013-supple.pdf]
